# Microscale tissue engineering of liver lobule models: advancements and applications

**DOI:** 10.3389/fbioe.2023.1303053

**Published:** 2023-12-08

**Authors:** Qi Wang, Juan Liu, Wenzhen Yin, Anqi Wang, Jingjing Zheng, Yunfang Wang, Jiahong Dong

**Affiliations:** ^1^ Department of Hepatobiliary and Pancreatic Surgery, The First Hospital of Jilin University, Jilin University, Changchun, China; ^2^ Research Unit of Precision Hepatobiliary Surgery Paradigm, Chinese Academy of Medical Sciences, Beijing, China; ^3^ Hepato-Pancreato-Biliary Center, Beijing Tsinghua Changgung Hospital, School of Clinical Medicine, Tsinghua University, Beijing, China; ^4^ Institute for Organ Transplant and Bionic Medicine, Tsinghua University, Beijing, China; ^5^ Key Laboratory of Digital Intelligence Hepatology, Ministry of Education, School of Clinical Medicine, Tsinghua University, Beijing, China; ^6^ Clinical Translational Science Center, Beijing Tsinghua Changgung Hospital, Tsinghua University, Beijing, China

**Keywords:** liver lobule model, 3D bioprinting, microfluidics, biomaterials, biomimic

## Abstract

The liver, as the body’s primary organ for maintaining internal balance, is composed of numerous hexagonal liver lobules, each sharing a uniform architectural framework. These liver lobules serve as the basic structural and functional units of the liver, comprised of central veins, hepatic plates, hepatic sinusoids, and minute bile ducts. Meanwhile, within liver lobules, distinct regions of hepatocytes carry out diverse functions. The *in vitro* construction of liver lobule models, faithfully replicating their structure and function, holds paramount significance for research in liver development and diseases. Presently, two primary technologies for constructing liver lobule models dominate the field: 3D bioprinting and microfluidic techniques. 3D bioprinting enables precise deposition of cells and biomaterials, while microfluidics facilitates targeted transport of cells or other culture materials to specified locations, effectively managing culture media input and output through micro-pump control, enabling dynamic simulations of liver lobules. In this comprehensive review, we provide an overview of the biomaterials, cells, and manufacturing methods employed by recent researchers in constructing liver lobule models. Our aim is to explore strategies and technologies that closely emulate the authentic structure and function of liver lobules, offering invaluable insights for research into liver diseases, drug screening, drug toxicity assessment, and cell replacement therapy.

## 1 Introduction

The liver, the largest digestive organ in the human body, also holds the distinction of being the most critical organ for maintaining internal homeostasis (Gebhardt and Matz-Soja, 2014). It boasts a repertoire of over 1,500 functions encompassing metabolism, secretion, excretion, and various biotransformation processes (Trefts et al., 2017). The human liver is composed of numerous structurally identical and similarly sized units known as liver lobules. These liver lobules represent the fundamental architectural and functional units of the liver and exhibit a complex microenvironment that can be broadly categorized into two components: cells and extracellular matrix (ECM).

The creation of a biomimetic liver lobule microenvironment is a prerequisite for constructing *in vitro* liver lobule models. Precision cell patterning techniques are of paramount importance in tissue engineering, as the accurate positioning of parenchymal and NPCs to recapitulate the intricate natural architecture of the liver tissue is a primary challenge in liver tissue engineering (Allen and Bhatia, 2002; Andersson and van den Berg, 2004). Cultivating one or more types of NPCs together with hepatocytes within a specific ECM and replicating the positioning of microvilli according to their authentic structure allows hepatocytes to acquire a specific cellular microenvironment, enabling the establishment of models with distinct functionalities. The construction of 3D liver lobule models offers advantages for hepatocytes, including the preservation of their natural morphology, diffusion gradients, long-term viability, and liver function (Guguen-Guillouzo and Guillouzo, 2010; Lubberstedt et al., 2011; Cipriano et al., 2017; Jin et al., 2021).

Over the past two decades, with the emergence and advancement of new technologies such as microfabrication (Zhao et al., 2023), photolithography (Hsieh et al., 2010), 3D bioprinting (Janani et al., 2022; Kim et al., 2023), and microfluidics (Ya et al., 2021), the biomimetic fidelity of liver lobule models has progressively increased. The latter two technologies, as cutting-edge liver simulation techniques, are capable of accurately replicating the structure and functionality of the liver, thus providing an efficient and dependable experimental platform for liver disease research and therapeutic development. 3D bioprinting allows the programmed assembly of different cell types with a wide range of bioinks and is ideally suited for the reproduction of liver lobule structures. Microfluidics exposes hepatocytes to fluid flow, emphasizing the dynamic simulation of the liver lobule microenvironment (e.g., nutrient exchange, shear stress on blood flow through the hepatic sinusoids, etc.). The two are not mutually exclusive, and their combined application can further advance the realistic reproduction of *in vitro* hepatic lobular models.

**FIGURE 1:**
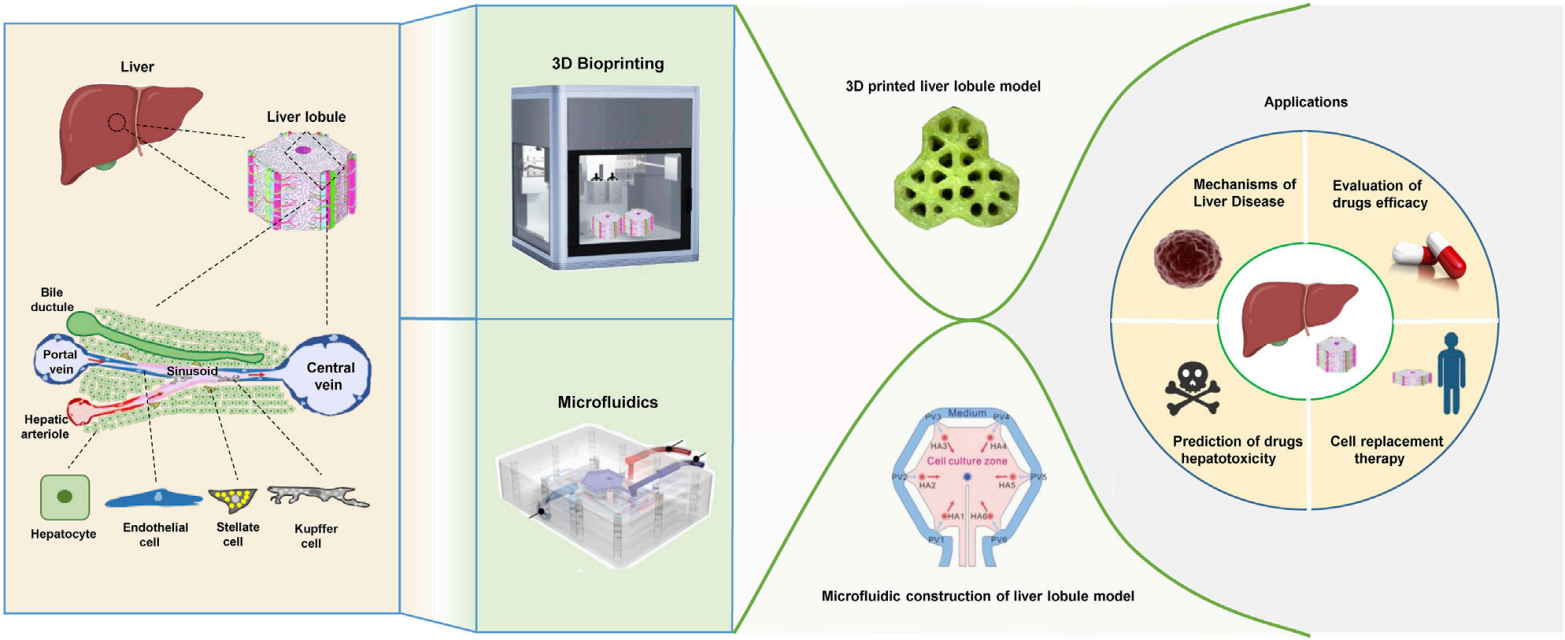
The physiological structure of liver lobules and the techniques, morphological structures, and applications of the liver lobule model. (The figure of microfluidics was adapted with permission from (Ya et al., 2021), Copyright © 2021, American Chemical Society; The figure of 3D printed liver lobule model was adapted with permission from (Khati et al., 2022), Creative Commons Attribution 4.0 International License; The figure of microfluidic assembly of liver lobule model was adapted with permission from (Du et al., 2021), Copyright © 2021, Acta Materialia Inc. Published by Elsevier Ltd.).

This review focus on the development of liver lobule model structures through the utilization of 3D bioprinting and microfluidic systems. It systematically summarizes the microenvironment factors and cells, construction methodologies, and specific morphological distinctions applied in the construction of liver lobules. The aim is to explore the optimal strategies for constructing biomimetic liver lobules, with the ultimate goal of providing a valuable platform for liver disease modeling, drug hepatotoxicity assessment, drug screening, and cell replacement therapy (Figure 1).

## 2 Physiological structure and microenvironmental composition of liver lobules

Liver lobule is structured as a hexagonal framework composed of a terminal hepatic vein “central vein,” hepatic plates, hepatic sinusoids, and bile canaliculi. These elements radiate outward from the central vein to form portal vein tracts in the periphery, structures within these tracts include bile ducts and ductules, hepatic artery, portal vein, lymphatic vessels, nerve fibers, and a few inflammatory cells. Hepatic sinusoids formed by microvessels travel between hepatocytes and, between the liver sinusoidal endothelial cells (LSECs) and hepatocytes, Disse spaces containing connective tissue (collagen type Ⅲ) forming the reticulin framework, and hepatic stellate cells (HSCs), facilitating the exchange of substances between blood and hepatocytes (Trefts et al., 2017; Janani et al., 2022). Together, the above structures form the unique spatial layout of the liver lobules.

The liver is composed of approximately 60%–70% liver cells, the parenchymal cells responsible for the liver’s primary functions, including the regulation of substance metabolism (e.g., bile synthesis, lipid metabolism, glucose metabolism, hormone metabolism, etc.), Detoxification and defence (conversion of endogenous or exogenous toxic substances into non-toxic or less toxic substances) and bile secretion (release of various insoluble bilirubins into the bloodstream) (Chen et al., 2020; Kumar et al., 2021). The remaining 30%–40% of liver cells constitute the non-parenchymal cell (NPC) population, which encompasses a diverse array of cells, each with distinctive roles and functions (Knook and Sleyster, 1980). Among these NPCs, LSECs play a critical role. LSECs form the inner lining of hepatic sinusoids, serving as a structural scaffold for hepatocytes. Their fenestrated endothelium allows substances to freely diffuse between the bloodstream and hepatocyte surfaces. In addition, LSECs have the ability to scavenge denatured macromolecular lipids (Elvevold et al., 2004), endocytose and scavenge certain ECM components such as hyaluronic acid (Hansen et al., 2005), and present antigens (Onoe et al., 2005), and also secrete biologically active factors and ECM components that are integral to the maintenance of hepatocyte function. LSECs are in a quiescent state under normal conditions. When the liver is subjected to injury such as inflammation or mechanical stimulation, LSECs are activated and their phenotype changes from quiescent to activated. Activated LSECs participate in the formation of hepatic fibrosis and the reconstruction of intrahepatic structures through proliferation and secretion of collagen and extracellular matrix components such as glycoproteins and proteoglycans on the one hand, and increase intrahepatic sinusoidal pressure through cell contraction on the other. In addition, LSECs store vitamin A and lipids, synthesise matrixmetalloproteinase (MMP), express cytokines and receptors such as hepatocyte growth factor (HGF) (DeLeve and Maretti-Mira, 2017; Guo et al., 2022).

The Disse space is situated between LSECs and hepatocytes. Within this space, the sinusoidal lumen is filled with plasma from the blood sinusoids, and the microvilli of the hepatocytes are immersed in it. This region serves as a crucial site for substance exchange between hepatocytes and the bloodstream. Additionally, this space hosts lipid-storing cells, also known as HSCs, characterized by their irregular morphology and the presence of 1–14 lipid droplets, about 1.0–2.0 μm in diameter, rich in vitamin A and byproducts, and triglycerides within their cytoplasm. HSCs perform various essential functions, including the uptake and storage of vitamin A for maintaining vision, promoting bone growth and development, protecting skin, as well as the synthesis of extracellular matrix and fibers. In cases of chronic liver disease, HSCs undergo abnormal proliferation and progressively transform into fibroblasts, a process closely associated with the development of hepatic fibroproliferative lesions. This pathological transformation plays a significant role in the progression of liver diseases (Wisse et al., 1985). Kupffer cells (KCs) function in phagocytosis, collagen synthesis, and cytokine secretion. Natural killer cells (NKs) participate in immune regulation within the NPCs repertoire (Utoh et al., 2018). This intricate interplay among hepatocytes and the various NPCs types highlights the liver’s exceptional functional complexity, underscoring its pivotal role in sustaining physiological homeostasis. Given this, several studies on LTE aim to reconstruct the components and structure of the Disse space (Lee-Montiel et al., 2017; Li et al., 2018).

The liver lobule’s microenvironment exhibits characteristics of metabolic zonation, with heterogeneity among different regions of hepatocytes. As blood flows directionally through the hepatic sinusoids from the PV area to the central vein, hepatocytes take up oxygen, nutrients and metabolise hormones, generating a gradient along the periportal-pericentral axis to shape the microenvironment. In turn, this gradient is one of the main drivers of differential gene expression and functional heterogeneity of cells within the lobules (Cunningham and Porat-Shliom, 2021). Thus, the PV zone, characterized by high oxygen levels and abundant nutrients, primarily handles functions requiring high energy demand, including oxygen uptake, glucose transport, gluconeogenesis, urea synthesis, fatty acid oxidation, and cholesterol synthesis. In contrast, the central venous (CV) zone, with lower oxygen levels, mainly deals with functions requiring lower energy demand, such as glucose uptake, glycolysis, amino acid synthesis, bile acid production, and lipid synthesis. The transitional zone in between plays a role in iron regulation and other functions (Mitani et al., 2017; Ben-Moshe and Itzkovitz, 2019; Manco and Itzkovitz, 2021). Spatial metabolic compartmentalisation allows opposite metabolic functions to operate simultaneously.

ECM is a complex three-dimensional network secreted by specific cells in tissues and organs. The liver ECM comprises insoluble complexes of collagens (fibrillar and network), adhesion proteins (e.g., laminin, fibronectin) and proteoglycans (PGs) (e.g., heparan sulfate PGs, chondroitin sulfate PGs). It not only provides structural support but also plays a critical role in various aspects, including cellular communication, growth regulation, and the regulation of tissue-specific gene expression (Reid et al., 1992; Janani and Mandal, 2021). And when the hepatic microenvironment is stimulated by various factors, the composition of the ECM can be dynamically regulated as it interacts with liver cells (Martinez-Hernandez and Amenta, 1993). The simulation of native liver-specific ECM can be achieved through decellularized liver matrix, which essentially restores the native characteristics of the natural ECM (Pati et al., 2014) (Figure 1).

## 3 Biomaterials and cells used to construct liver lobule models

Whether it is 3D bioprinting or microfluidic systems, the application of biomaterials and cells aims to faithfully replicate the authentic liver lobule microenvironment and structure. High-quality biomaterials used to construct the liver lobule microenvironment not only generate spatial scaffolds resembling the *in vivo* conditions for cells but also simulate the interactions between cells and the extracellular matrix microenvironment. They can further provide cells with chemical and mechanical stimuli akin to those found in the body, thus maintaining or enhancing cellular functions (Bissell and Choun, 1988; Arriazu et al., 2014). Meanwhile, complementing the liver lobule microenvironment are hepatocytes forming the hepatic parenchyma, endothelial cells creating the hepatic sinusoids connecting the portal field to the central vein, KCs residing within the hepatic sinusoids, hepatic stellate cells situated between hepatocytes and endothelial cells, and specific specialized ECM (Utoh et al., 2018; Khati et al., 2022).

### 3.1 Biomaterials for the mimicking of liver lobule microenvironment

While the microenvironment surrounding hepatocytes is crucial for the establishment and enhancement of their function, the majority of current research does not focus on constructing liver lobule microenvironments. Conventional methods involving simple culture media or biomaterials solely serving as support remain the primary means of rapidly and inexpensively creating liver lobule microenvironments, but they are no longer the prevailing trend. For instance, regarding the culture medium, the use of the same medium as employed in 2D cell cultures is a common approach. As for biomaterials, on one hand, the choice often revolves around the use of natural biomaterials (e.g., Gelatin Methacrylate (GelMA) (Ma et al., 2016), Collagen (Hong et al., 2021), Gelatin (Ya et al., 2021), Fibrinogen (Du et al., 2021), Agarose (Macdonald et al., 2018), Alginate (Yajima et al., 2018; Zheng et al., 2019)), which exhibit strong cell compatibility and offer advantages such as ease of handling and remolding. However, these materials tend to have reduced mechanical properties, limited availability, and faster degradation rates. GelMA, compared to regular gelatin, offers superior biological functionality and tunable mechanical properties. It is employed in the construction of structurally stable 3D bio-scaffolds with customizable degradation characteristics (Klotz et al., 2016). Collagen, a crucial component of the ECM, is among the preferred materials for liver tissue engineering applications. It boasts excellent biocompatibility, biodegradability, and bioactive sites supporting cell adhesion, growth, and differentiation. Due to its relatively weaker mechanical properties and higher degradation rate, it is often used as an auxiliary factor to enhance the biocompatibility of bioinks (Vasanthan et al., 2012; Benwood et al., 2021). Alginate, a negatively charged polysaccharide, possesses non-toxicity, hydrophilicity, good biocompatibility, and the ability to cross-link with multivalent cations like Ca^2+^ (Ye et al., 2019). At room temperature, alginate exhibits low mechanical strength, inferior biocompatibility compared to natural protein-based biomaterials, and limited adhesive properties (Ye et al., 2019; Piras and Smith, 2020). Hence, modifying its properties or utilizing it in combination with other materials represents the primary strategies for overcoming these limitations (Maji et al., 2023; Zhang et al., 2023). On the other hand, synthetic biomaterials (Polyethylene glycol diacrylate (PEGDA) (Cui et al., 2018)) offer advantages like high mechanical strength, excellent flexibility, strong processability, and tunable degradation rates. Nevertheless, they often lack cell recognition sites, exhibit poor cell adhesion, and may have limited biocompatibility (Gungor-Ozkerim et al., 2018). The advantages of polyethylene glycol lie in its excellent biocompatibility, hydrophilicity, non-toxicity, and non-immunogenicity. It is commonly incorporated as a hydrophilic component into poly (lactic acid) molecules, forming amphiphilic block copolymers (D'Souza A and Shegokar, 2016).

In addition to the aforementioned biomaterials used in constructing liver lobule models, there are other suitable biomaterials for 3D bioprinting. Among natural biomaterials, silk fibroin stands out due to its high resolution and excellent biological properties, such as flexibility, tensile strength, and biocompatibility. It holds great potential in tissue engineering. Sharma et al. developed a bioink for liver tissue engineering using silk fibroin-gelatin-dECM that significantly enhances the liver-specific genes expression of hepatocytes (Kundu et al., 2013; Sharma et al., 2021). Chitosan, known for its flexibility, non-toxicity, biodegradability, and cell affinity, is particularly suitable for low-temperature shaping. Nanofiber scaffolds made from chitosan modified with surface lactose ligands not only exhibit high printing stability but also enhance the viability of primary hepatocytes (Feng et al., 2009). Among synthetic biomaterials, poly (lactic acid), known for its good biocompatibility, mechanical strength, transparency, and heat resistance, has been used to provide a favorable nanofiber scaffold microenvironment for the *in vitro* regeneration of primary rat hepatocytes (Bierwolf et al., 2011). Polyvinyl alcohol, an important water-soluble polymer, has been used by Khati et al. as sacrificial material to provide channels for perfusion, supporting the development of densely populated liver models (Khati et al., 2022). Poly (ε-caprolactone), a high-molecular-weight polymer with good biocompatibility and biodegradability, has been utilized by Salerno et al. to develop a 3D vascularized liver tissue based on biodegradable hollow fiber membranes (Salerno et al., 2020). However, it is difficult for a single biomaterial to meet the physical and biochemical requirements for constructing a liver lobule model, so a decellularized ECM (dECM)-based multicomponent bioink would be a good choice for simulating the liver lobule microenvironment (Kim et al., 2022). Advancements in tissue engineering techniques have enabled the precise removal of liver cells, resulting in decellularized scaffolds that retain the complex three-dimensional structure and extracellular matrix components of the organ. It possesses excellent biocompatibility, low immunoreactivity, and physical and chemical properties akin to natural tissues (Zhang et al., 2022). They are prepared using decellularization technology, which removes cellular or nuclear components (including antigenic components that can induce immune inflammation) while preserving the structural and functional proteins of the ECM. This provides an ideal environment for cell adhesion, growth, and differentiation (Allu et al., 2023).

Progressively, in order to provide a better growth environment for hepatocytes, researchers often use methods of indirect biological secretion or direct supplementation to introduce nutritional components into the support materials (3D printing) or culture media (3D printing and microfluidics). For instance, many researchers co-culture hepatocytes with murine embryonic fibroblast cells—NIH/3T3, which provide nutrients through growth factor secretion, within a 3D environment, significantly enhancing hepatocytes functionality (Cui et al., 2018; Zheng et al., 2019; Wu et al., 2020; Khati et al., 2022). Furthermore, the direct addition of ECM components is a crucial method for supporting hepatocytes. Decellularized liver matrix can almost entirely mimic the natural liver lobule microenvironment. Notably, Guagliano, G. et al. designed a hybrid alginate-ECM specifically for flexible simulation of the liver environment *in vitro*. Researchers used freeze-dried and powdered porcine liver ECM to provide suitable physiological and biochemical support for the implanted cells. Moreover, the alginate component can modulate cross-linking dynamics. This biomaterial has been shown not only to promote cell proliferation but also to facilitate the formation of cellular aggregates (Marshall et al., 2021; Im et al., 2022; Guagliano et al., 2023). Taking it a step further, researchers have incorporated other crucial cell factors and components into the culture system, including BA silk fibroin, RGD motifs, β-D Galactose, and decellularized porcine liver ECM. These scaffolds were designed to support vascularization within the liver lobule model structure and enhance hepatocyte functionality (Janani et al., 2022). Consequently, it is evident that a foundation based on comprehensive ECM functionality, augmented with specific cell factors, will be a pivotal direction for future liver lobule microenvironment construction.

### 3.2 Cells for constructing liver lobule models

Currently, most liver lobule models involve a limited variety of cellular components. For instance, Macdonald et al. employed a novel coplanar Dielectrophoresis (DEP) system to pattern a single human liver cancer cell line, HepG2/C3A, into a liver lobule model. Despite the presence of a single cell component, their research results demonstrated a significant enhancement in liver-specific functionality, such as albumin secretion, compared to non-patterned cells (Macdonald et al., 2018). Models containing two types of cellular elements are the most common type. Typically, co-cultivation of HepG2 and NIH/3T3 as mentioned earlier (Cui et al., 2018; Zheng et al., 2019; Wu et al., 2020; Khati et al., 2022). Alternatively, models can incorporate HepG2 cells with endothelial cells, including human umbilical vein endothelial cells (HUVECs), Human umbilical vein endothelial cell fusion cells–EA.hy926, or Bovine carotid artery normal endothelial cells (HH cells), to simulate liver lobule structures (Ho et al., 2006; Ho et al., 2013; Yajima et al., 2018; Kang et al., 2020; Hong et al., 2021). For instance, Wu, Y. et al. conducted co-cultivation of HepG2 with NIH/3T3 cell lines utilizing microextrusion (ME) -based bioprinting techniques, leading to the establishment of robust hepatocellular functionality, as observed in their study (Wu et al., 2020). Jin, S. et al., on the other hand, utilized a preset extrusion bioprinting approach with a microfluidic emulsification system to construct biomimetic liver lobules in high throughput, demonstrating significant albumin secretion, urea production, and CD31 expression (Kang et al., 2020; Hong et al., 2021). Some researchers have employed a combination of three cell types, including the assembly of human induced pluripotent stem cell-derived hepatic progenitor cells (hiPSC-HPCs), HUVECs, and Adipose-derived stem cells (ADSCs) through 3D bioprinting. HUVECs and ADSCs were chosen to represent support cells from the endothelial and mesenchymal lineages, as they possess both primitive and angiogenic potential (Baranski et al., 2013; Takebe et al., 2013; Takebe et al., 2014; Ma et al., 2016). Additionally, others have utilized a combination of Human adipose mesenchymal stem cell (hAMSC)-derived hepatocyte-like cells (HLCs), HUVECs, and Human HSCs. Furthermore, microfluidic assembly techniques have been employed to construct liver lobule model structures by combining HepaRG cell lines, HHSCs, and LSECs in conjunction with the fibrinogen solution. In the scenario with the most diverse cell types, Ya, S. et al. obtained four distinct cell components–Hepatocytes, LSECs, HSCs, and KCs–from the livers of 4∼6-week-old BALB/c mice. These cells were isolated using collagenase digestion and flow cytometry identification. Alongside collagen, they constructed a structure closely resembling the liver lobule microenvironment (Ya et al., 2021). Li et al. recreated the 3D structure of liver sinusoids using a three-layer microfluidic device with primary human hepatocytes, primary LSECs, LX2 cells and KCs. The authors utilized primary LSECs as the lining of vascular channels, reproducing partial immunologic functions within the liver sinusoid. This led to the activation of LSECs, promotion of polymorphonuclear leukocytes (PMNs) binding, followed by transmigration into the hepatic chamber (Li et al., 2018).

Regardless of the number of cell types applied, hepatic parenchymal cells are indispensable, and there are currently four main sources of hepatic parenchymal cells for constructing hepatic lobular microstructures, namely, primary human hepatocytes (Li et al., 2018), primary mouse hepatocytes (Ya et al., 2021; Kim et al., 2023), Human adipose mesenchymal stem cell-derived hepatocyte-like cells (Janani et al., 2022), hiPSC-HPCs (Ma et al., 2016), HepG2 (Khati et al., 2022), and HepaRG (Du et al., 2021). As the ''Gold Standard'', primary hepatocytes possess the morphological characteristics of normal hepatocytes and faithfully reflect the markers and functions expected of these cells in the living organism. Consequently, they provide a more authentic representation of *in vivo* physiological functions, making them particularly well-suited for exploring physiological mechanisms, and assessing drug toxicity and understanding of mechanisms responsible for hepatotoxicity (disruption of cellular energy status, alteration of Ca^2+^ homeostasis, inhibition of transport systems, metabolic activation, oxidative stress, covalent binding, etc.) (Gomez-Lechon et al., 2010). Therefore, primary hepatocytes are instrumental in establishing highly realistic liver lobule models. LeCluyse et al. elaborated methods for the isolation of primary human hepatocytes from liver tissue obtained from an encapsulated end wedge removed from patients undergoing resection for removal of liver tumors or resected segments from whole livers obtained from multiorgan donors, methods for culturing these primary hepatocytes in various matrix compositions and geometries (LeCluyse et al., 2005). Moreover, the evolving technology of primary hepatocyte preservation can maintain their viability and metabolic activity in a high-throughput and low-toxicity manner for metabolic studies and toxicity tests (Hengstler et al., 2000; de Vries et al., 2019). Meanwhile, there are some obstacles to the use of primary hepatocytes. To optimize the survival and growth of primary liver cells, additional nutrients need to be supplemented beyond those provided by traditional culture media. Despite these efforts, primary hepatocytes have limited passage numbers *in vitro*, making their application costly (Ramboer et al., 2014). Furthermore, the majority of primary hepatocytes used for constructing liver lobule models are derived from animals, and there is a stringent ethical support for Human-derived hepatocytes (Ya et al., 2021; Kim et al., 2023). Immortalized liver cell lines adapt well to the culture environment, are easy to cultivate, and offer high yields. However, compared to primary hepatocytes, these cell lines typically exhibit genetic and phenotypic differences from their source cells, and sometimes even undergo morphological changes. These disparities make it challenging to replicate many physiological and pathological processes, ultimately hindering the faithful reconstruction of liver lobule functionality (Zeilinger et al., 2016; Kvist et al., 2018; Huggett et al., 2022). hiPSC-HPCs offer distinct advantages over traditional cells, including their human origin, ease of access, scalability, avoidance of ethical concerns related to human embryonic stem cells, and the potential for developing personalized medicine using patient-specific iPSCs (Shi et al., 2017). However, compared to primary hepatocytes, it also faces many challenges in producing iPSC-derived cells for Liver tissue engineering. Ehrlich et al. indicate that immature iPSC-derived hepatocytes typically have less metabolic capacity and consume only 25% of the oxygen (Ehrlich et al., 2019). It is worth mentioning that due to the lack of sufficient cell sources and associated immune responses for liver tissue engineering, patient PSC-derived cells have significant potential for use in the construction of hepatic lobules containing a wide range of cell types. Gough et al. have summarised the challenges in producing iPSC-derived cells, noting that apart from no protocols have yet been reported for the generation of LSECs, other cell types such as Hepatocyte-like cells Cholangiocyte-like cells, Hepatic stellate-like cells, Macrophage-like (Kupffer) cells, and so on, have shown remarkable progress (Gough et al., 2021). hAMSCs are readily accessible and abundant in source, exhibiting greater stability in their cell properties during culture compared to other stem cells. They are considered a safe and reliable option with a robust differentiation potential, capable of directed differentiation into functional cells of various systems under specific conditions. Furthermore, they do not experience a decline in activity with age (Bunnell, 2021). However, similar to iPSC-derived cells, hepatocytes derived from this cell source are also difficult to highly restore the function of primary hepatocytes. So, it is less used in the construction of liver lobule model.

In conclusion, concerning biomaterials and cell types, they represent profoundly critical factors influencing the functionality and structures of liver lobule model. Precisely arranging and depositing diverse biomaterials and cells within a 3D environment, promoting cell-cell and cell-ECM interactions, can provide a highly promising platform for disease modeling, drug screening, and other applications (Mazzocchi et al., 2018) (Table 1).

**TABLE 1:** Liver lobule traits.

Techniques	Year	Methodologies	Fine shape	Internal structure	Size	Bioinks (% w/v)	Activity, phenotype, function	Ref.
3D bioprinting-based liver lobular engineering	2020	Preset EBB with a microfluidic emulsification system	Hollow cylindrical cord	Hollow concentric sectors composed of hepatocytes spaced by endothelial cells	Cross-sectional diameter: ∼1 mm	HepG2/C3A; EA.hy926; Alginate (3%); Neutralized collagen (3%)	Cell viability (>90%); Albumin: +; CYP3A4: +	Kang et al. (2020)
	2021	Preset EBB with a microfluidic emulsification system	Spheroids or cylindrical cord	Concentric sectors composed of hepatocytes spaced by endothelial cells	Spheroids diameter or cylindrical cord cross-sectional diameter: 416 ± 26.14 μm	HepG2/C3A; EA.hy926s; Highly viscous atelocollagen-based bioink (4% collagen hydrogels)	Cell viability (>80%); Albumin: +; Urea: +; CYP3A4: +	Hong et al. (2021)
	2022	Dual tube EBB	Multi-layer triangle	Sectors composed of hepatocytes spaced by endothelial cells	Side length: ∼2 cm	hAMSC-derived HLCs; HUVECs; HHSCs; BA silk fibroin; RGD motif; β-D Galactose; Liver ECM; Gelatin	Day 15 vs. Day 1: Cell proliferation: #; Albumin: ##; Urea: ##; LDH: ###	Janani et al. (2022)
	2022	Dual tube EBB	Three connected skeletonized hexagons	Hepatocytes and endothelial cells are arranged parallel along the fan-shaped skeleton	Single hexagon diameter: ∼8 mm	HepG2; NIH/3T3; dLM-PEG-T; Polyvinyl alcohol (sacrificial support material)	Cell viability (>90%); Day7 vs. Day 1: Albumin: #; MKI67: #	Khati et al. (2022)
	2016	Sequential input photo-assisted bioprinting	Multiple connected skeletonized hexagons (honeycomb-like)	Concentric sectors composed of hepatocytes spaced by endothelial cells	Single hexagon diameter: ∼1 mm. Thickness: ∼200 μm	hiPSC-HPCs; HUVECs; ADSCs; GelMA (5%, for hiPSC); GelMA (2.5% for supporting cells); GMHA (1% for supporting cells)	Albumin: +; Urea: +; CYP3A4: +	Ma et al. (2016)
	2020	EBB	Multiple connected skeletonized hexagons (honeycomb-like)	Supporting cells surround the hepatocytes in a circular pattern	Single hexagon diameter: ∼3 mm	HepG2; NIH/3T3; Alginate (1%); CNC (3%); GelMA (5%)	Day 14 vs. Day 1: Cell area: ##; Albumin: ####	Wu et al. (2020)
	2023	EBB	Skeletonized Circle	Hepatocytes arranged in a circular pattern with a cross in the center	Diameter: ∼11.5 mm	HepG2; Hep3Gel: a hybrid alginate−ECM hydrogel	Day 12 vs. Day 3: Cell proliferation: ##	Guagliano et al. (2023)
	2023	EBB	Three connected skeletonized hexagons	Hollow concentric sectors composed of hepatocytes spaced by endothelial cells	Single hexagon diameter: ∼1 mm	Primary mouse hepatocytes (PMHs); HUVECs; dECM gBioink (dECM- gelatin composite bioink)	Cell viability (>80%); Albumin: +; Urea: +	Kim et al. (2023)
Microfluidic assisted liver lobular engineering	2021	Microfluidic guided liver lobular chip with hepatic sinusoid network	Single planarized hexagon	Seven regularly arranged CV/PV/HA structures	Diameter: ∼7 mm	Collagen; Hepatocytes; LSECs; HSCs; KCs. (The primary liver cells were isolated from mice); Gelatin (0.25%)	Flow rates: 50 μL/min vs. 25 μL/min: Cell viability (>80%); Number of Sinusoids: ++++; CYP3A4: +++; ALB: +++	Ya et al. (2021)
	2021	Microfluidic guided liver lobular chip with dual blood supply	Single planarized hexagon	Six HA channels surrounded by PV channels with CV channels in the center	Diameter: ∼2 mm	HepaRG; LX2 cells (human HSCs); HHSECs; Fibrinogen solution; Thrombin	Cell viability (>80%); Albumin: ++; Urea: +; CYP3A4: ++	Du et al. (2021)
	2006	Enhanced field-induced DEP trap	Single planarized concentric circles	Hepatocytes and endothelial cell cords arranged at radial intervals	Diameter: ∼1 mm	HepG2; HUVECs	Cell viability (>90%)	Ho et al. (2006)
	2013	Enhanced field-induced DEP trap	Multiple planarized hollow hexagons	Hepatocytes and endothelial cell cords arranged at radial intervals	Single hexagon diameter: ∼2.5 mm	HepG2; HUVECs	Cell viability (>90%); CYP450-1A1: +	Ho et al. (2013)
	2018	Coplanar DEP	Multiple planarized hollow hexagons	Hepatocyte cords are arranged radially	Single hexagon diameter: ∼800 μm	HepG2/C3A; Agarose	Cell viability (>70%); Albumin: +	Macdonald et al. (2018)
	2018	DMD-based microfluidic channel	Hollow 6-tooth gear-like cords	Hollow gear-like structures of hepatocytes mixed with supporting cells	Cross-sectional diameter: ∼1 mm	HepG2; NIH/3T3; PEGDA hydrogel	Cell viability (>90%); Day 15 vs. Day 1: Cell spreading: ####; Cell proliferation: ####; Albumin: +; Urea: +	Cui et al. (2018)
	2019	PIED	Hollow 6-tooth gear-like cords	Hollow gear-like structures of hepatocytes mixed with supporting cells	Cross-sectional diameter: ∼1 mm	HepG2; NIH/3T3; Alginate	Cell viability (>90%); Albumin: +; Urea: +	Zheng et al. (2019)
	2018	Sandwich type fiber cores made by microfluidic devices	Multiple circular cords	Bilateral empty hepatocytes surrounded by endothelial cells	Single cord cross-sectional diameter: ∼150 μm	HepG2; HH cells (bovine carotid artery normal endothelial cell); NaAlg	Cell viability (>80%); Albumin: ++; CYP3A4: +	Yajima et al. (2018)
	2017	Microfluidic system consisting of glass, Polydimethylsiloxane and plastic	Cuboid	Pipeline structure	Plane of oxygen measurement (80 mm above the cell layer); Pipeline structure diameter: ∼10 μm	Primary human hepatocytes; Human dermal microvascular endothelial cells (HMVEC-D); EA.Hy926; LX2 cells; KCs (from THP-1 and U937 cell lines); Collagen; LECM; Fibronectin	Albumin: +; Urea: +; CYP2E1: +	Lee-Montiel et al. (2017)
	2018	Microfluidic devices predominantly composed of glass	Cuboid	Three layered devices	3 glass layers A1–A3 (15 mm × 45 mm)	Primary human hepatocytes; LX2 cells; Primary LSECs; KCs (from THP-1 cell lines); Collagen; Porcine LECM.	Albumin: +; Urea: +	Li et al. (2018)

**Notes:** Improvement magnitude in the experimental group compared to the control group: 0∼1x: +; 1∼3x: ++; 3∼10x: +++; >10x: ++++; Improvement magnitude in the comparisons of time series: 0∼1x: #; 1∼3x: ##; 3∼10x: ###; >10x: ####. **Abbreviations:** EBB: extrusion-based bioprinting; HA: hepatic arteriole; CV: central venous; dLM-PEG-T: dLM crosslinked with succinimidyl valerate-polyethylene glycol-succinimidyl valerate and mushroom tyrosinase; CNC: cellulose nanocrystal; GelMA: gelatin methacryloyl; PMHs: Primary mouse hepatocytes; DEP: dielectrophoresis; DMD: digital micromirror device; PIED: Photo-induced electrodeposition; PEGDA: polyethylene glycol diacrylate; NaAlg: Alginate polymer, conjugated with GRGDSP peptide; LDH: lactate dehydrogenase.

## 4 3D bioprinting techniques applied to construct liver lobule models

**FIGURE 2:**
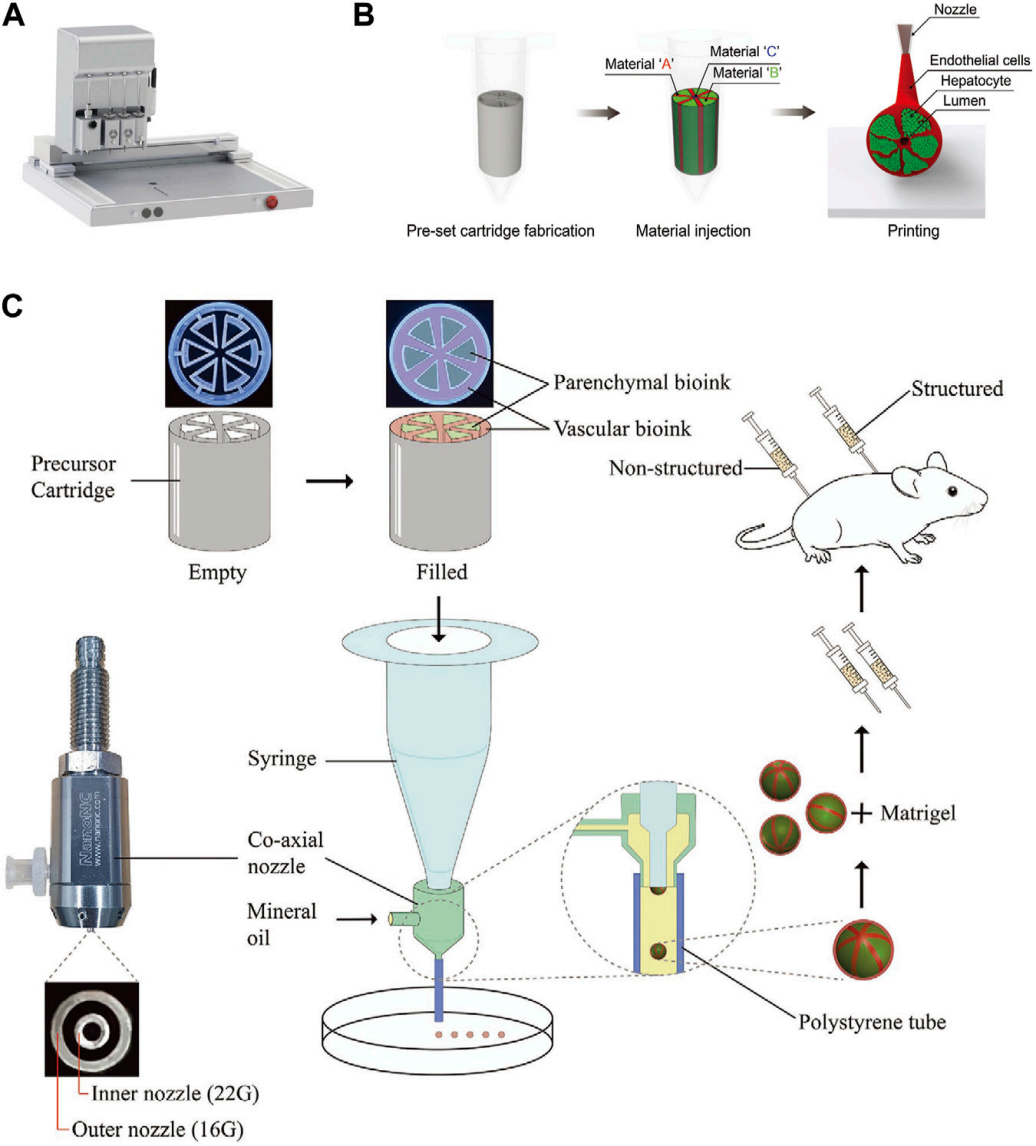
The 3D bioprinting techniques used to construct liver lobule models. **(A)** The “PanoSpace BioPro” 3D bioprinter form Beijing Panospace Biotech Co., Ltd. **(B)** Preset extrusion bioprinting with a microfluidic emulsification system (Adapted with permission from (Kang et al., 2020), copyright © 2020, WILEY-VCH Verlag GmbH & Co. KGaA, Weinheim). **(C)** Schematic representation of the research strategy. Microtissue spheroids are fabricated by combining preset extrusion bioprinting with a microfluidic emulsification system. The microtissue spheroids with or without biomimetic structure are used for the *in vivo* Matrigel plug assay. (Adapted with permission from (Hong et al., 2021), copyright © 2021, Wiley-VCH GmbH).

3D bioprinting technology offers the advantage of precise patterning of cells and biomaterials, making it a valuable tool for creating increasingly complex liver lobule models. Various 3D bioprinting methods, such as inkjet-based bioprinting (IBB), extrusion-based bioprinting (EBB), and photo-assisted bioprinting, have been developed. The choice of printing head depends on factors like temperature control, synchronized photopolymerization, and co-axial dual-component printing. Utilizing biocompatible, low-immunogenicity, low-toxicity, and highly hydrophilic biomaterials in conjunction with tools such as electrostatic direct-writing printing plates, printing spindles, and multi-aperture printing nozzles, various structures of different shapes, sizes, and material compositions can be rapidly and efficiently printed (Faulkner-Jones et al., 2015; Ma et al., 2018; Matai et al., 2020; Tully and Meloni, 2020; Saggiomo, 2022). Currently, *in vitro* liver lobule models are mainly done by EBB technology, which uses pneumatic/piston/screw-driven syringe pump to extrude bioink and is able to print temperature-dependent gels by controlling the print head, but it suffers from problems such as low resolution and easy clogging of the nozzles (Ozbolat and Hospodiuk, 2016; Ma et al., 2018; Willson et al., 2020) (Figure 2A); IBB is suited to print low-viscosity biomaterials, which are fast to fabricate, low-cost, and high-resolution, but cannot print at high cell densities (Xu et al., 2005; Gu et al., 2020; Nie et al., 2020); Photo-assisted bioprinting can print complex and fine structures with the highest precision, but it faces the problem of cellular phototoxicity and inability to create horizontal gradients in the structure (Melchels, Feijen, and Grijpma, 2010).

### 4.1 Progress in 3D bioprinting liver lobule models

The development of 3D-printed liver models has rapidly advanced from single-cell to multi-cell printing, from amorphous to biomimetic structures, from process-oriented to functional designs, and from single techniques to interdisciplinary approaches (Ma et al., 2020; Sun et al., 2023). Firstly, it is important to emphasize that printing biomaterials directly into scaffolds for cell adhesion, culture and functional construction via 3D bioprinting is an excellent way to go. This method has significant advantages over printing bioinks with cellular components, including better print resolution and minimal cytotoxicity during printing (Salerno et al., 2019; Wang, 2019). However, it has not been used in large numbers for tissue engineering of liver lobules, which may be attributed to the poorer establishment of liver lobule function. Currently, 3D-printed liver lobule models mainly consist of hepatocytes co-cultured with NPCs (Ma et al., 2016; Janani et al., 2022), multi-cell and different ECM mixed-printing (Wu et al., 2020), and liver tissues of actual size with vascularized structures (Kang et al., 2020). These liver lobule model exhibited higher liver function performance compared to simple 3D cultures. Several published works have been accomplished using these 3D bioprinting technologies. Notably, Jin, S. et al. have creatively utilized a preset extrusion-based bioprinting system with a microfluidic emulsification approach to preassemble bio-inks according to their positional relationships before extrusion, allowing for the efficient fabrication of spherical or fibrosis cord structures with liver lobule-like cross-sectional microarchitecture. This method is both fast and yields uniform dimensions (Kang et al., 2020; Hong et al., 2021) (Figures 2B, C). It represents an innovative approach in the field of 3D printing.

### 4.2 Designing liver lobule models by 3D bioprinting

As mentioned earlier, the intricate internal structure of the liver lobule expands the total surface area for blood flow, facilitating the functionality of cells. Therefore, constructing an idealized liver lobule model involves central endothelial components with a hollow center, radiating endothelial cells that separate hepatocytes, and finally encapsulating the entire structure with endothelial cells. This design allows for the separate simulation of the CV zone, sinusoids, and PV zone. The faithful reconstruction of the liver lobule structure represents the fundamental and critical first step towards the success of liver tissue engineering.

**FIGURE 3:**
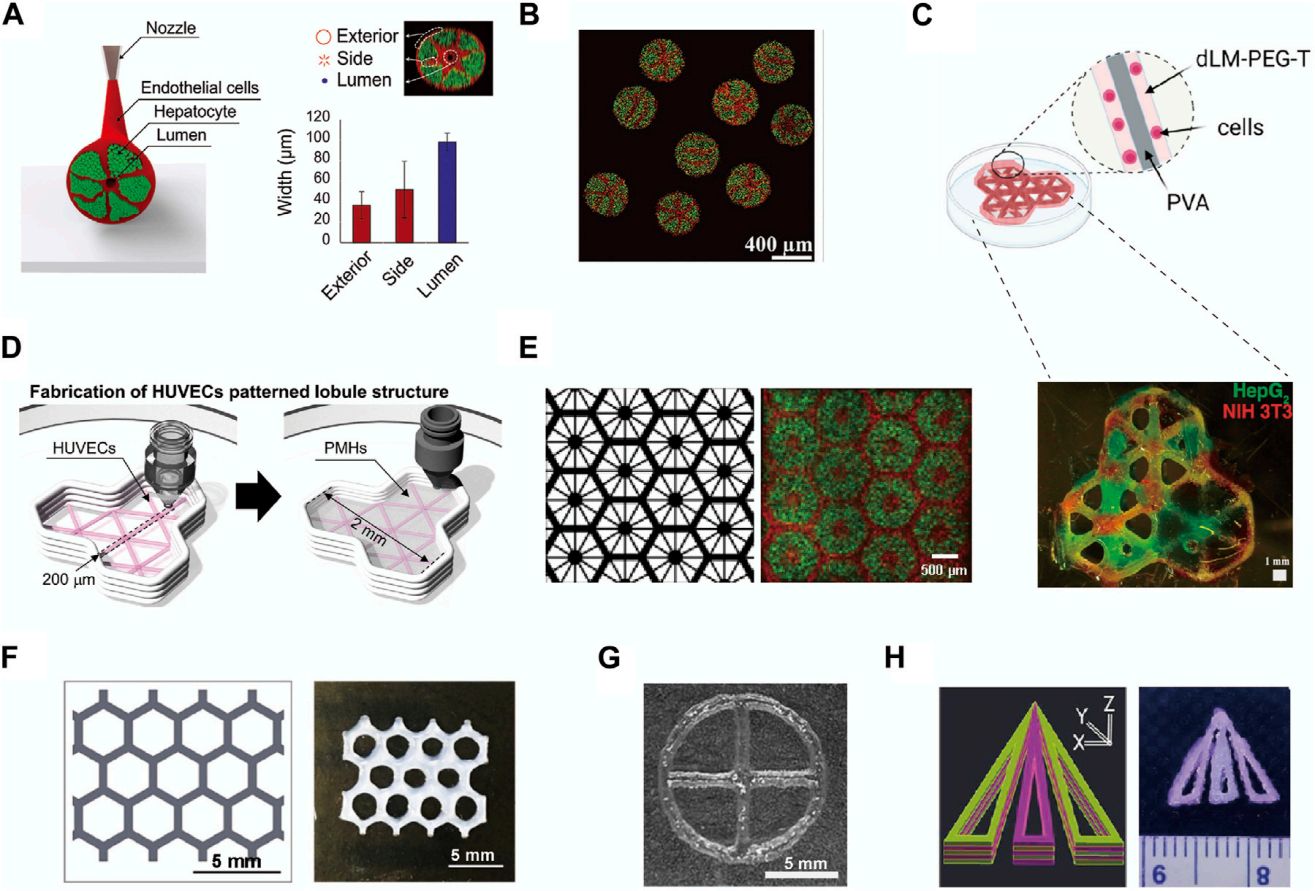
The fine structure of liver lobule models constructed by 3D Printing. **(A and B)** The structure of cord-shaped or spherical liver lobule models constructed using preset extrusion bioprinting. (Adapted with permission from (Kang et al., 2020), copyright © 2020, WILEY-VCH Verlag GmbH & Co. KGaA, Weinheim; (Hong et al., 2021), copyright © 2021, Wiley-VCH GmbH). **(C)** 3D printing of a polyvinyl alcohol trilobular structure. (Adapted with permission from (Khati et al., 2022), Creative Commons Attribution 4.0 International License.). **(D)** Pattern diagram for 3D printing to produce a model of a trilobular structure. (Adapted with permission from (Kim et al., 2023), Creative Commons Attribution 4.0 International License.). **(E)** Images showing patterns of grayscale digital masks (left) and fluorescently (right) labeled hiPSC-HPCs (green) and supporting cells (red). (Adapted with permission from (Ma et al., 2016), Creative Commons Attribution 4.0 International License.). **(F)** Schematic of the liver lobule-mimetic honeycomb structure. (Adapted with permission from (Wu et al., 2020), Creative Commons Attribution 4.0 International License.). **(G)** Liver lobule model printed with Hep3Gel biomaterials. (Adapted with permission from (Guagliano et al., 2023), Creative Commons Attribution 4.0 International License.). **(H)** Schematic illustration of computer-aided design designing and a multilayered biomimetic liver lobule model. (Adapted with permission from (Janani et al., 2022), Copyright © 2022, American Chemical Society).

3D bioprinting offers a high degree of shape controllability for liver lobule model structures. In theory, with the selection of suitable materials and bioink compositions, it is possible to 3D print liver lobule models of various shapes within certain size constraints. Additionally, multi-head printing ensures material complexity. After meticulous research into the precise shapes of numerous liver lobule models, they can be broadly categorized as follows. From an overall structural perspective, these categories include: Hollow cylindrical cord (Kang et al., 2020) (Figure 3A); Spheroids (Hong et al., 2021) (Figure 3B); Three connected skeletonized hexagons (Khati et al., 2022) (Figures 3C, D); Multiple connected skeletonized hexagons (honeycomb-like) (Ma et al., 2016; Wu et al., 2020) (Figures 3E, F); Skeletonized circle (Guagliano et al., 2023) (Figure 3G); Trilobal triangle (Janani et al., 2022) (Figure 3H) and so on. From the internal arrangement (cross-section) within the liver lobule models, Songwan’s team has designed a model that closely mimics the authentic liver lobule structure. In the cross-section, a hollow core is enveloped by a ring of endothelial cells. Radiating outward from this core are six cell cords, with the gaps between the cords filled with hepatocytes. The entire structure is further surrounded by an outer ring of endothelial cells (Kang et al., 2020) (Figure 3A). Other types of structures, when compared to the model described above, exhibit slight variations, including Concentric fan-shaped structures composed of hepatocytes spaced by endothelial cells (Ma et al., 2016; Hong et al., 2021) (Figures 3B, E); Hepatocytes and supporting cells arranged parallel along the fan-shaped skeleton (Khati et al., 2022) (Figures 3C, D); Supporting cells surrounding hepatocytes in a circular pattern (Wu et al., 2020) (Figure 3F); Hepatocytes arranged in a circular pattern with a cross in the center (Guagliano et al., 2023) (Figure 3G); and Skeletonized triangles composed of hepatocytes spaced by skeletonized triangular endothelial cells (Janani et al., 2022) (Figure 3H). By summarizing the impact of the aforementioned structural designs on the functionality of liver lobule models, we regretfully observe that these designs predominantly emphasize enhancing the viability and functionality of hepatocytes (e.g., albumin secretion, urea synthesis) rather than focusing on achieving metabolic zonation and vascular flow in liver lobule models (Table 1).

## 5 Microfluidic techniques applied to construct liver lobule models

The development of a cell alignment and deposition system based on microfluidic perfusion technology has enabled us to dynamically and efficiently fabricate various liver lobule model structures. Coupled with advanced tissue engineering techniques, this approach can simulate authentic microenvironments effectively (Rennert et al., 2015).

### 5.1 Innovative applications of microfluidic techniques in liver lobule modelling

**FIGURE 4:**
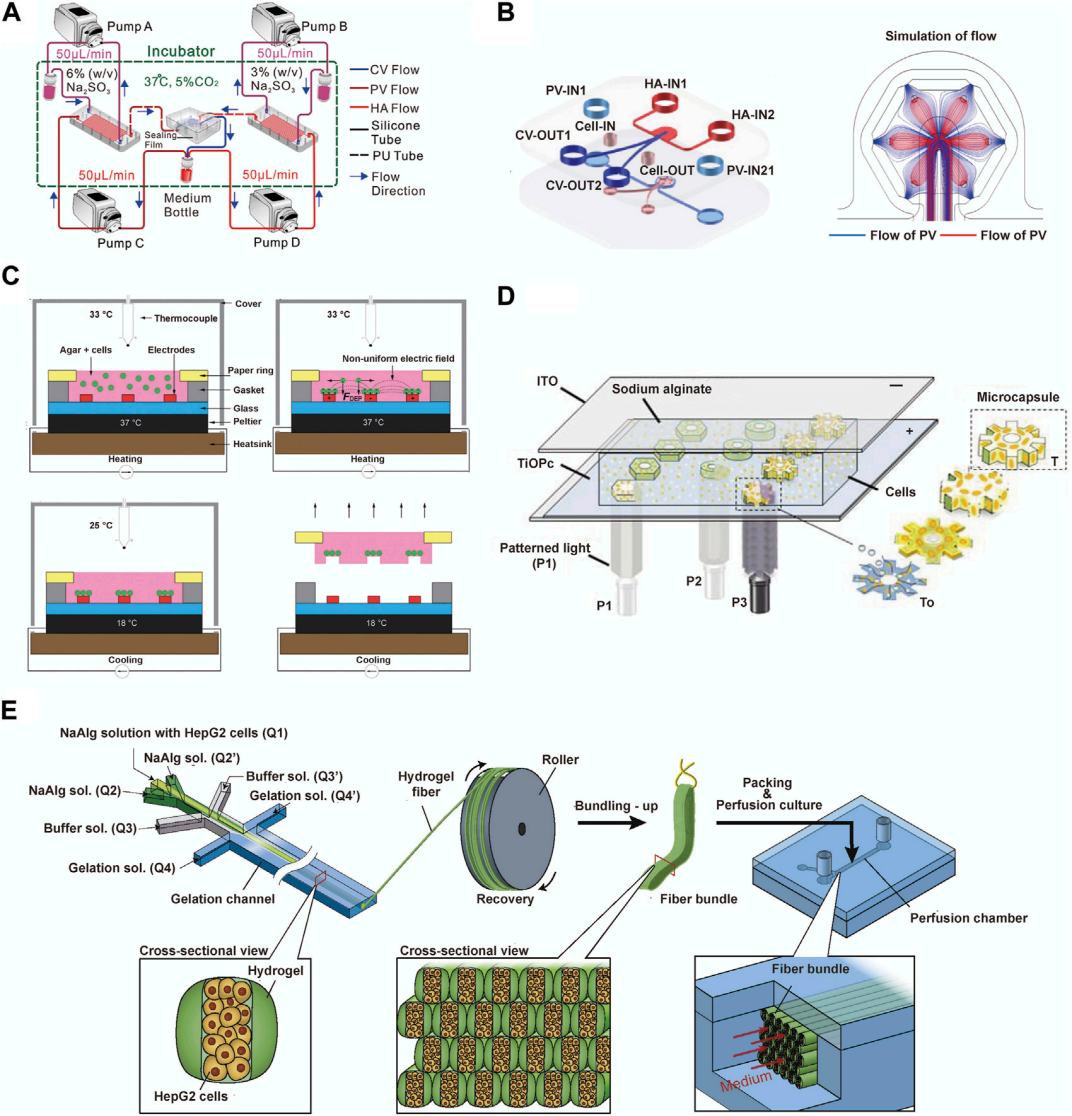
The microfluidics techniques used to construct liver lobule models. **(A)** Experiment setup for the liver lobule chip. In the operation of the liver lobule chip, all samples were collected from the medium bottle (red) using a micropipettor. (Adapted with permission from (Ya et al., 2021), Copyright © 2021, American Chemical Society). **(B)** The fabrication procedures and the simulated flow of the liver lobule chip. (Adapted with permission from (Du et al., 2021), Copyright © 2021, Acta Materialia Inc. Published by Elsevier Ltd.). **(C)** The ''build and transfer'' system setup and operation for patterning liver lobule structures within an agar gel with a paper substrate. (Adapted with permission from (Macdonald et al., 2018), Creative Commons Attribution 3.0 International License.). **(D)** Photo-induced electrodeposition system. (Adapted with permission from (Zheng et al., 2019), Copyright © 2019, American Chemical Society.). **(E)** Schematic showing the bundling-up assembly of the cell-laden hydrogel microfibers. (Adapted with permission from (Yajima et al., 2018), Copyright © 2018, The Society for Biotechnology, Japan.).

The microfluidic liver lobule chip is an organ-on-chip model of the liver manufactured using microfluidic technology. The primary advantage of microfluidic systems lies in their ability to dynamically simulate and monitor, transporting cells or other culture components to specified locations, completing the input and output of culture media under the control of micro-pumps, and providing cells with pressure, shear forces, oxygen, and nutrients (Abdellatef et al., 2014; Rennert et al., 2015; Ramadan and Zourob, 2020). The design of microfluidic chips based on perfusion is more common. Ya, S. et al. presented a flow-guided perfusable angiogenesis approach to liver lobule chip production, creating a radial hepatic sinusoid network. And it was complemented by an oxygen concentration regulating chip (ORC) designed to provide physiologically precise dissolved oxygen concentrations necessary for the actual generation of hepatic arterioles and venules, thus faithfully replicating the microenvironment of the liver lobule (Ya et al., 2021) (Figure 4A). Further, Li et al. created a vascularised human liver acinus microphysiological system (vLAMPS) equipped with upper hepatic and lower vascular channels using a dual-channel glass device. The system is separated by a 3 mm aperture polyethylene terephthalate membrane and recreates the physiological zonation of the liver lobules by generating an oxygen gradient through the consumption of oxygen by hepatocytes. Currently, vLAMPS is rapidly evolving towards the use of all patient-specific primary cells for modelling (Li et al., 2018). Additionally, Du, K. et al. designed a dual blood supply liver lobule chip that offers dynamic flow through portal vein (PV) and hepatic arteriole (HA) to support various types of cells (Figure 4B).

Another significant advantage of microfluidic technology is its integration with advanced systems designed to serve specific functions. DEP is a biomanipulation technique applied in microfluidic chips and molecular separation detection technologies. It enables the translational motion of neutral particles situated in a non-uniform electric field due to dielectric polarization, facilitating the orderly arrangement of cells (Henslee, 2020; Waheed et al., 2021). Ho, C.T. et al. designed a chip featuring an enhanced field-induced DEP trap. This chip can separately manipulate hepatocytes and endothelial cells initially randomly distributed within microfluidic chambers and arrange them into patterns mimicking the morphology of liver lobules (Ho et al., 2006; Ho et al., 2013). Similarly, Macdonald, N.P. et al. developed a chip based on novel coplanar DEP, integrating temperature control with a Peltier cooler, laser-cut paper rings, and cell patterning within a hydrogel to create a reusable liver lobule model. However, a limitation of this system is its inability to assemble cells separately based on cell type (Macdonald et al., 2018) (Figure 4C). Digital micromirror devices (DMDs) are devices that use digital voltage signals to control the mechanical movements of micro mirrors, thus achieving optical functions. The device consists of a liquid reservoir that holds a material capable of curing under specific wavelength ultraviolet (UV) light exposure. The imaging system is positioned beneath the reservoir, with its imaging surface precisely located at the bottom of the liquid reservoir. Through energy and graphical control, it is possible to cure a certain thickness and shape of material with each exposure, gradually building up a 3D solid by layer-by-layer exposure and elevation. The operation of a DMD involves a coordinated process integrating light, mechanics, and electronics. To meet specific functional requirements, coordinated control of the optical path, micro mirror movement, and circuitry is essential (Mott et al., 2016; Cui et al., 2018). Fukuda, T. et al. mixed hydrogel with cellular components and photopolymerized them into gear-shaped patterns within microfluidic channels based on DMD. The liver lobule design with luminal structures provides a natural advantage for drug perfusion and toxicity testing (Cui et al., 2018). Building upon this foundation, the team further utilized a photo-induced electrodeposition (PIED) system to fabricate liver lobule models in various shapes (Zheng et al., 2019) (Figure 4D), offering insights for generating more complex multicellular structures in future tissue engineering. Additionally, a sandwich-type hydrogel microfiber fabrication system has been developed to create microtissue fibers, where the core encapsulates cells. The obtained fibers are bundled through recycling using a roller and packaged into microfluidic perfusion chambers for cultivation (Yajima et al., 2018) (Figure 4E).

### 5.2 Diverse structural variations in microfluidic liver lobule models

**FIGURE 5:**
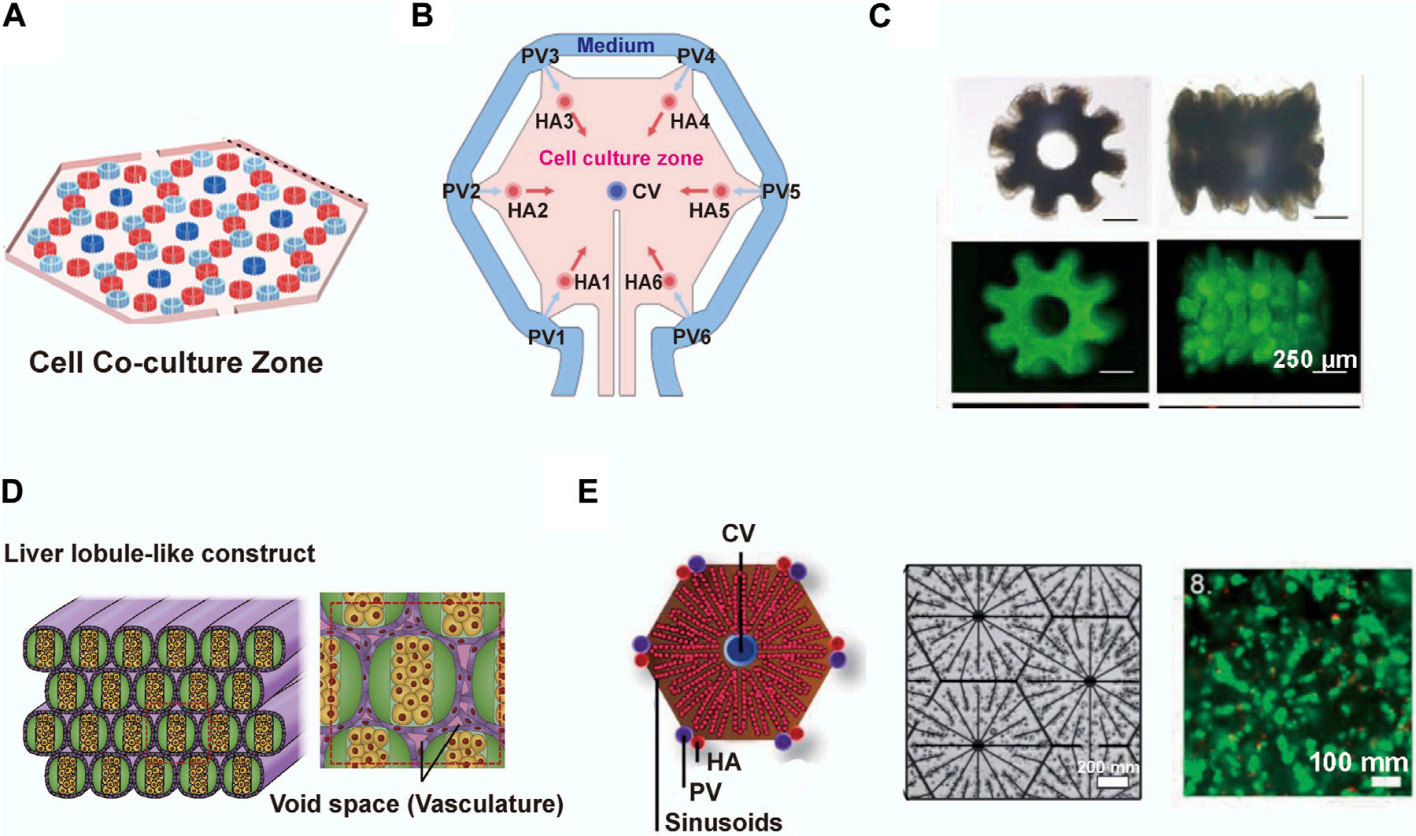
The detailed structure of liver lobule models constructed by microfluidic techniques. **(A)** The schematic components of the liver lobule model containing CV/PV/HA. (Adapted with permission from (Ya et al., 2021), Copyright © 2021, American Chemical Society). **(B)** A liver lobule chip with dual blood supply. (Adapted with permission from (Du et al., 2021), Copyright © 2021, Acta Materialia Inc. Published by Elsevier Ltd.). **(C)** Top and side views of released microtissues generated by micropillar-guided assembly and conventional assembly. (Adapted with permission from (Zheng et al., 2019), Copyright © 2019, American Chemical Society.). **(D)** Vascular network-like structures using fibers covered with endothelial cells. (Adapted with permission from (Yajima et al., 2018), Copyright © 2018, The Society for Biotechnology, Japan.). **(E)** The schematic diagram (left) and the microscopic image (right) illustrate HepG2/C3A cells being immobilized *in situ* by DEP forces, forming a liver lobule model. (Adapted with permission from (Macdonald et al., 2018), Creative Commons Attribution 3.0 International License.).

The structural diversity of liver lobule models constructed using microfluidic technology relies on both the direct design of the chip and the indirect influence of auxiliary technique. When examining the overall structural appearance, these models include: Single planarized hexagon (Du et al., 2021; Ya et al., 2021) (Figures 5A, B); Hollow 6-tooth gear-like cords (Cui et al., 2018; Zheng et al., 2019) (Figure 5C); Multiple circular cords (Yajima et al., 2018) (Figure 5D) and so on. From the perspective of the internal arrangement (cross-section) within the liver lobule models, they encompass: Multiple regularly arranged CV/PV/HA structures (Ya et al., 2021) (Figure 5A); Six HA channels surrounded by PV channels with CV channels in the center (Du et al., 2021) (Figure 5B); Hollow gear-like structures of hepatocytes mixed with supporting cells (Cui et al., 2018; Zheng et al., 2019) (Figure 5C); Bilateral empty hepatocytes cluster surrounded by endothelial cells (Yajima et al., 2018) (Figure 5D); Hepatocytes or endothelial cell cords arranged at radial intervals (Ho et al., 2006; Ho et al., 2013; Macdonald et al., 2018) (Figure 5E). Compared to 3D bioprinting, microfluidics offer distinct advantages in achieving the microscale functionalities of liver lobules, such as oxygen gradients, metabolic zonation, and vascular flow. For example, as mentioned earlier, Ya et al. have developed a microfluidics-guided vascularization approach to construct perfusable liver sinusoid network models. This model can simulate the physiological concentration gradients provided by actual hepatic arterioles and venules by modulating oxygen levels. Such liver lobule designs yield more biomimetic liver microstructures, higher metabolic capacity, and prolonged hepatocyte functionality (Ya et al., 2021) (Figures 4A, 5A) . Regrettably, in liver lobule models involving microfluidic technology, there is a notable scarcity of models that construct metabolic zonation with heterogeneous hepatocytes. The majority still predominantly emphasize vascular flow (Table 1).

## 6 Future and prospects

Liver-related diseases constitute a major cause of illness and death globally, characterized by their diversity and complex etiology, and for the most part, they lack effective treatment options (Siegel et al., 2022). The development of liver models has made significant contributions to the understanding of the cellular biology and biochemistry of both normal and pathological liver states, particularly in substance metabolism and toxicity (Guguen-Guillouzo and Guillouzo, 2010). The realization of liver function relies on the interaction between hepatocytes and the liver microenvironment. Therefore, the construction of *in vitro* liver models primarily aims to emulate the liver microenvironment, providing a promising platform for comprehending liver physiology and pathology mechanisms, drug development, pre-clinical drug toxicity assessment, and even cell replacement therapy for liver transplant patients.

The liver lobule, measuring approximately 1 mm in size, represents a fundamental functional unit within the liver. It comprises various components, including the hepatic sinusoid, Disse’s space, bile ducts, hepatocytes, LSECs, KCs, and stellate cells, some other immune cells and ECM (Moragas et al., 1992; Crawford, Lin, and Crawford, 1998). The intricate architectural arrangement of the liver lobule poses a significant challenge for tissue engineering. While some studies have successfully generated liver lobule models containing four distinct cell types, these models lack the ECM support and realistic cellular arrangement, leaving further potential for enhancement in terms of functional reconstruction (Ya et al., 2021). Another critical factor in constructing liver lobule models is the zonation of hepatocytes. We believe that, based on the elucidation of characteristic gene expression in the PV/CV zone hepatocytes, it is a promising strategy to create specific heterogeneous liver cells through genetic engineering and then use tissue engineering techniques for spatial reintegration, thereby achieving metabolic zonation differences.

3D bioprinting enables precise deposition of biomaterials to create specific microenvironments, including cell-cell and cell-ECM interactions that are lacking in 2D cell culture systems. This approach allows for the construction of models *in vitro* for disease modeling and drug screening that surpass conventional cell and animal models in terms of cost and time efficiency (Mazzocchi et al., 2018; Leucht et al., 2020; Szklanny et al., 2021). Microfluidic chips can faithfully simulate the flow conditions of blood *in vivo*, providing a liver lobule microenvironment with controllable O_2_ and nutrient gradients. These chips are characterized by high repeatability, stability of results, and efficiency (Yang et al., 2022). Technological advancements are driving liver lobule models towards achieving 1:1 anatomical microstructure replication, although there is still a considerable gap to be bridged.

It is important to note that today, 3D bioprinting and microfluidics are no longer two separate ways of constructing liver lobule models, but both can be used together to create liver microphysiological systems (MPS). Liver MPS, also known as liver microarrays, are miniaturised functional units of the liver constructed by self-assembly or 3D-supervised placement using multiple cellular components. It emphasises the reduction of the physical and biochemical microenvironment of the liver lobule, such as, structural position, microfluidic flow, intercellular communication, and phenotypic function (Gough et al., 2021; Low et al., 2021).

Future directions in the development of liver lobule models include improving the relevance of the microenvironment to the real liver lobule, refining the dimensions of the fabricated structures, and enhancing the control of shape and structure, all of which are aimed at reproducing 3D models with robust mechanical properties and biological functions.

## 7 Conclusion

This study focuses on the construction of liver lobule models, with an emphasis on replicating the authentic liver lobule microenvironment and cell arrangement. It summarizes the current mainstream techniques for preparing liver lobule models, specifically the biological components utilized in 3D bioprinting and microfluidics, as well as the detailed technical methods and structures of the models. This work provides guidance for future efforts to construct models that closely mimic the real liver lobule microenvironment. Furthermore, it offers a promising platform for research on liver-related diseases, drug evaluation for liver diseases, prediction of drug-induced liver toxicity, and the potential analysis of cell replacement therapy for liver transplant patients.

## Figures and Tables

**Table:** 

**ECM**	extracellular matrix
**LSEC**	liver sinusoidal endothelial cell
**HSC**	hepatic stellate cell
**NPC**	non-parenchymal cell
**KC**	Kupffer cell
**MMP**	matrixmetalloproteinase
**HGF**	hepatocyte growth factor
**NK**	natural killer cell
**PEGDA**	Polyethylene glycol diacrylate
**DEP**	Dielectrophoresis
**HUVEC**	human umbilical vein endothelial cell
**ME**	microextrusion
**hiPSC-HPC**	human induced pluripotent stem cell-derived hepatic progenitor cell
**ADSC**	adipose-derived stem cell
**hAMSC-derived HLC**	human adipose mesenchymal stem cell -derived hepatocyte-like cell
**PMNs**	polymorphonuclear leukocytes
**IBB**	inkjet-based bioprinting
**EBB**	extrusion-based bioprinting
**CV**	central venous
**ORC**	oxygen concentration regulating chip
**vLAMPS**	vascularised human liver acinus microphysiological system
**PV**	portal vein
**HA**	hepatic arteriole
**DMD**	digital micromirror device
**UV**	ultraviolet
**PIED**	photo-induced electrodeposition
**dLM-PEG-T**	dLM crosslinked with succinimidyl valerate-polyethylene glycol-succinimidyl valerate and mushroom tyrosinase
**CNC**	Cellulose nanocrystal
**GelMA**	Gelatin methacryloyl
**PEGDA**	polyethylene glycol diacrylate. NaAlg: Alginate polymer, conjugated with GRGDSP peptide
**MPS**	microphysiological systems
